# Error Rates in Users of Automatic Face Recognition Software

**DOI:** 10.1371/journal.pone.0139827

**Published:** 2015-10-14

**Authors:** David White, James D. Dunn, Alexandra C. Schmid, Richard I. Kemp

**Affiliations:** 1 School of Psychology, The University of New South Wales, Sydney, Australia; 2 School of Psychology, The University of Sydney, Sydney, Australia; Bournemouth University, UNITED KINGDOM

## Abstract

In recent years, wide deployment of automatic face recognition systems has been accompanied by substantial gains in algorithm performance. However, benchmarking tests designed to evaluate these systems do not account for the errors of human operators, who are often an integral part of face recognition solutions in forensic and security settings. This causes a mismatch between evaluation tests and operational accuracy. We address this by measuring user performance in a face recognition system used to screen passport applications for identity fraud. Experiment 1 measured target detection accuracy in algorithm-generated ‘candidate lists’ selected from a large database of passport images. Accuracy was notably poorer than in previous studies of unfamiliar face matching: participants made over 50% errors for adult target faces, and over 60% when matching images of children. Experiment 2 then compared performance of student participants to trained passport officers–who use the system in their daily work–and found equivalent performance in these groups. Encouragingly, a group of highly trained and experienced “facial examiners” outperformed these groups by 20 percentage points. We conclude that human performance curtails accuracy of face recognition systems–potentially reducing benchmark estimates by 50% in operational settings. Mere practise does not attenuate these limits, but superior performance of trained examiners suggests that recruitment and selection of human operators, in combination with effective training and mentorship, can improve the operational accuracy of face recognition systems.

## Introduction

In modern forensic and security practice, automatic face recognition software is often used to augment important identification processes. An increasingly common application of face recognition technology is known as one-to-many identification—whereby pattern matching algorithms are used to compare a single probe image to large databases of facial images [1]. This function can be used to protect against identity fraud when issuing national identity documents such as passports, immigration visas and driving licences—by improving detection of duplicate applications by the same individual [2]. Further, the recent proliferation of image-based evidence from CCTV and mobile devices entails that facial images are often an important source of evidence in criminal investigations. In forensic applications, face recognition software therefore enables police officers to use this image evidence to search large databases of known offenders [3, 4, 5]. Similar technology is also used to enhance user experience in popular social media platforms.

Although the accuracy of face recognition software has improved markedly over the past two decades [1, 6], it is important to note that Automatic Face Recognition (FR) systems do not yet live up to their name—they are not entirely *automatic*. Identification accuracy can be quite poor in cases where image capture conditions are not optimal [7] and where images of a face are captured several years apart [8]. To manage this uncertainty, in many applications algorithms present human users with a ‘candidate list’ displaying the highest matching images returned from a database and ranked in order of similarity to the probe image. It then falls to the human operator to review this candidate list and check for the presence or absence of matching identities [3, 4, 9]. Similar combinations of computer and human processing are also used in fingerprint identification systems [10, 11].

Standardised benchmarking tests of algorithm accuracy are administered by the US National Institute of Standards and Technology. The most recent Face Recognition Vendor Test (FRVT) reports accuracy of leading commercial “off-the-shelf” algorithms in one-to-many identification [1]. In a particularly challenging test, recent police “mugshots” were used to probe an historic database of 1.6 million mugshot images containing one or more target images of each identity. Leading algorithms performed with a high level of accuracy, returning matching images of the target identity in the top ten ranked matches for between 80% and 97% of targets, depending on the algorithm. Although performance did vary depending on the size of the database being searched, the effect was typically modest, suggesting that accurate searches are also feasible in nation-sized databases, such as those held by passport-issuing agencies. Importantly however, this method only estimates accuracy of the machine component of the total system, ignoring the impact of the decisions made by the human operators—who monitor image galleries containing the highest ranking matches [1]. This is an important limitation because psychological research has shown people make large numbers of errors when matching photos of unfamiliar faces.

In an early demonstration of this problem, Bruce and colleagues [12] constructed police ‘line-up’ arrays containing ten high-resolution images of young adult male subjects. Participants had to decide if a target face was present in the photo gallery, and if they were, to select the matching identity. Despite target and gallery images being taken only minutes apart, in full frontal pose and under similar lighting conditions, mean error rates on this task were 30%. Subsequent studies have replicated this basic finding across a variety of stimulus sets and task formats [13–16]. Error-prone performance is also observed in people who perform face matching as part of their daily work. For example, White and colleagues [17] tested matching accuracy of passport officers in their workplace. Despite extensive experience in face matching, these staff were no more accurate than a group of university students—on tasks that modeled decisions encountered in their daily duties.

In this paper we describe the first published test of human performance on FR ‘candidate lists’ that are sampled from operational conditions. The format of this task was specifically designed to emulate workflow of passport issuance officers using FR software to detect fraudulent passport applications. Target images were real passport application images that were used as probes in an FR one-to-many search of a very large database of passport images. The purpose of this one-to-many search in the passport issuance process is to detect existing passports that contain a picture of the applicant, but under a different name, which is indicative of a fraudulent application. This search returns ‘candidate lists’ of images with highest ranked similarity to the target according to a scoring metric computed by state-of-the-art FR software.

We tested participants’ ability to detect images of the target identity in these candidate lists, by inserting images of the applicant from previous applications. Because we had limited time to conduct tests, and our aim was to provide reliable measures of perceptual matching ability on this task, we inserted matching identities on 50% of candidate lists. This was a necessary divergence from the target prevalence rate in many forensic and security applications, where targets appear in candidate lists very rarely [2, 18]. In the first experiment we test a group of untrained student participants on the FR workflow. We then compare performance of control participants to two groups of Passport Officers who operate FR systems as part of their daily work. We refer to these groups as *Facial Review* and *Facial Examiner* staff; corresponding to the different roles that they perform in their work, and the different levels of training and selection that they have received [19]. *Facial review* staff perform many face matching decisions per day, but check FR candidate lists as part of a series of eligibility checks that make up the majority of processing. These passport officers have typically received a small amount of training when commencing employment, and perform a similar role to passport officers tested in previous studies of face matching ability [17]. On the other hand, *Facial Examiners* were part of a smaller set of highly trained staff who perform more detailed forensic-comparison of facial images. In recent studies, similar groups of professional examiners have been shown to have superior face identification abilities [20, 21].

## Experiment 1

In the experiments reported in this paper we measure human accuracy on a user interface based on output from commercial-off-the-shelf face recognition software (Cognitec DBScan 4.1.0 B3). In the most recent international benchmarking test of leading commercial face identification systems, this algorithm correctly returned a matching image in the top eight ranked matches in approximately 90% of cases, when searching a database of 1.6 million police mugshot images of adults [1]. To generate candidate lists that simulated the work of passport issuance officers, in the tests reported here the algorithm searched a large image database of Australian passport holders and returned the eight highest ranking images in the database. Australian passports are valid for up to 10 years, and so the task involved matching by identity across images captured many years apart. Previous research has shown that face aging makes unfamiliar face matching more difficult, increasing error by 20% when images are separated by more than a year [17, 22, 23] and so we expected error rates to be higher than in similar test using images taken on the same day [12].

To test whether their accuracy varied as a function of target face age, participants performed the task with an equal number of child, adolescent and adult applicants. The human face undergoes significant anatomical change during childhood, and the change is both quantitatively [24] and qualitatively [25] different to changes occurring during adulthood. Thus, we also predicted that the effect of face aging on face matching accuracy reported in previous studies would vary across the course of facial development.

### Ethics Statement

**Fig 1:**
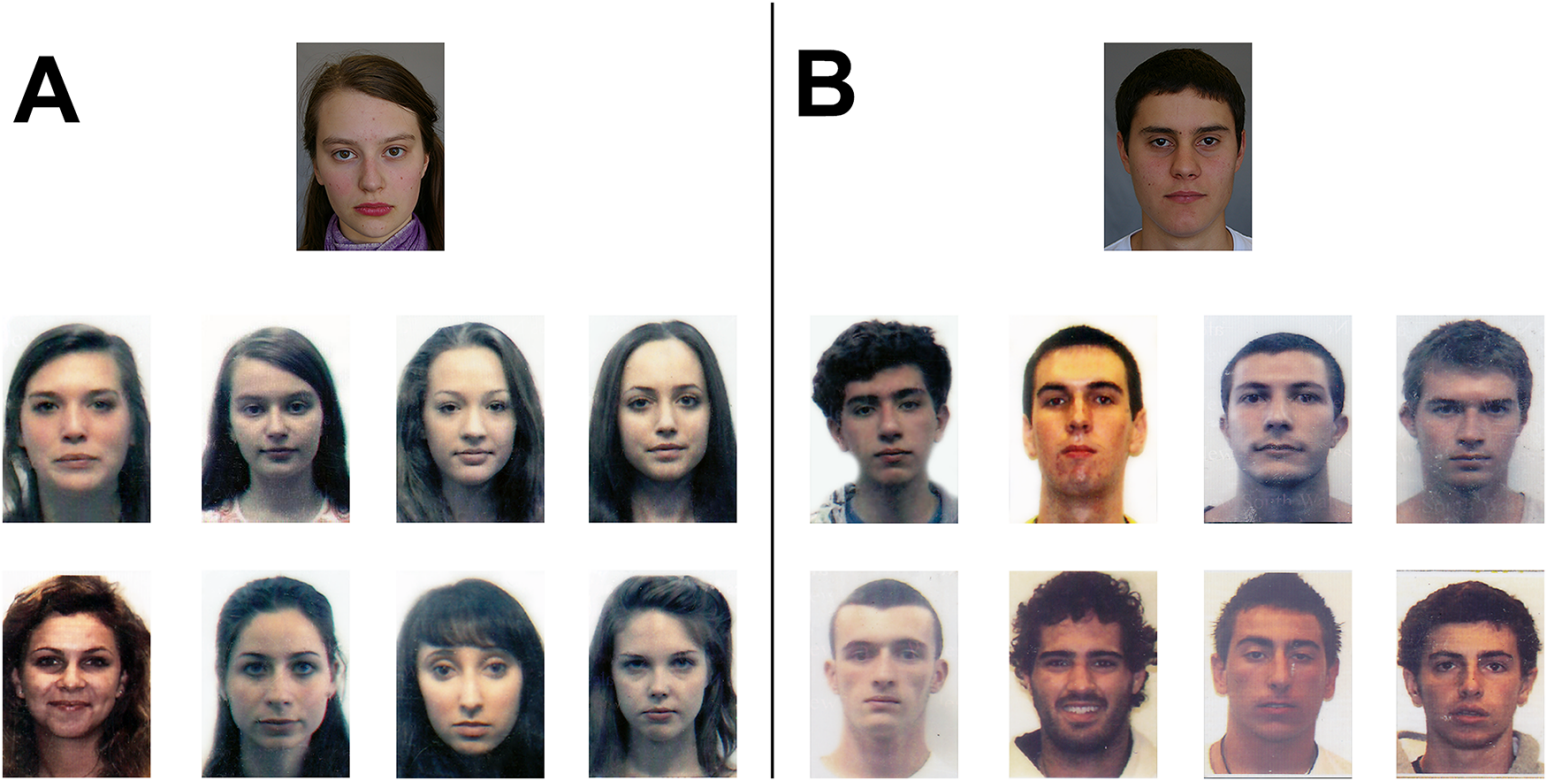
Example one-to-eight face matching arrays used to simulate FR ‘candidate lists’ in Experiments 1 and 2. Participants had to decide whether the target (top image) was present in the gallery below, and if so, select the matching identity (for solution to these examples, see Acknowledgements). Images are representative of the stimuli used in the matching task, but for reasons of privacy we are not able to provide examples of the passport images used in our studies.

This study was approved by the Human Research Ethics Committee at the University of New South Wales. All participants provided written informed consent and appropriate photographic release. The individuals shown in Fig 1 have given written informed consent (as outlined in PLOS consent form) to publish their image.

### Method

#### Participants

Forty-two undergraduate students (32 female) from the University of New South Wales participated in this experiment in exchange for course credit. Participants’ age ranged from 17 to 53 years, with a mean of 20.3 years (SD = 6.0). Six participants were excluded from our analysis because they did not finish the experiment in the allocated time of 1 hour and so final analyses were based on data from 36 participants (27 females).

#### Stimuli

Stimuli used in this study were images of Australian citizens that had consented for their photograph to be used in research during the passport application process. Images were sampled from a database containing passport application images of 20,000 anonymous identities who had at least one previous application image in the database. The database was a random sample of consenting Australian citizens and so demographic composition was reflective of Australian demographics (see S1 Data for item ethnicity and age). On the basis of meta-data generated by face recognition software, we excluded 5810 identities that were wearing glasses or had their mouth open. From the remaining set, we randomly sampled 408 child applicants, selected to have an age at application of between 6 and 13 years (Child condition; M = 10.0, SD = 2.0), 408 adolescent or young adult applicants, aged between 14 and 22 years (Adolescent condition; M = 18.2 SD = 2.65) and 408 adult applicants between 40 and 47 years (Adult condition; M = 43.4 SD = 2.30). An equal number of male and female applicants were sampled in each condition.

For each identity, a set of eight distractor identities were selected by the proprietary FR software. To simulate operational function of this software in an applied context, each application image was used as a probe to return the top eight ranked identities from the passport database (n > 1,000,000). These images were used as foil identities in the 1-to–8 candidate list arrays (see Fig 1). For target present trials, the foil with the lowest ranking match score was replaced by the image from that candidate’s previous application. In both target absent and target present trials, position of candidate images in the array was randomised. In Australia, the maximum validity of a passport is 5 years for minors (under 18 years), and 10 years for adults. As a result, the time interval between target image and the image from previous applications shown in the candidate list array varied as a function of condition. For Child applicants the mean was 6.2 years (SD = 1.7) for Adolescents it was 6.3 years (SD = 1.6) and for Adults it was 9.7 years (SD = 2.8).

#### Design and Procedure

The 1224 target identities were divided into four sets of 306 (consisting of 102 child, 102 adolescent, 102 adult applicants in each set) and each participant was randomly allocated to one of four sets for testing. We used this method to ensure that idiosyncratic properties of the age-defined sets did not confound effects of face age: averaging across many identities ensured that influences of identity-specific properties were ‘washed-out’. Each identity appeared once in the experiment, either in a target present or target absent trial (this was randomly determined for each subject). Each participant completed a total of 306 trials including 6 practice trials. Half of trials were target present and half were target absent. All images were presented at size 213 by 268 pixels on a 15” monitor with resolution 1920 by 1080 with each image subtending a visual angle of approximately 3 degrees (see Fig 1).

Participants were tested individually on laptop computers and the experiment was implemented in Psychtoolbox for Matlab [26]. Each participant was presented with 6 practice trials followed by 300 experimental trials. Practice trials were included to familiarise participants with response keys and so no feedback was provided on any trial.

On each trial, participants were presented with the photograph of the applicant (target face) and asked to decide if the target identity was present in the array and if they were present, to select the matching identity. On half of trials an image of the target face was present in the array and on the other half of trials the target was absent from the array, although this was not revealed to participants. Stimuli remained on the screen until the participant made a response and trial order was randomised for each participant. Trials were presented in three blocks of 100 trials with a short break provided between blocks. On average, the experiment lasted 50 minutes.

### Results

Overall percent correct was 45.1% (SD = 11.7%) for adult target images, 41.1% (SD = 11.7%) for adolescent target images and 39.0% (SD = 10.6%) for child target images. The main effect of applicant age on overall accuracy was significant [F (2,70) = 19.49, p < .05, η_p_^2^ = .358] with planned contrasts confirming lower accuracy in matching child and adolescent applicants compared to adult applicants [child V adult: t (35) = -6.87, *p* < .05, Cohen’s d = 1.17; adolescent V adult: t (35) = -4.06, p < .05, Cohen’s d = 0.68; child V adolescent: t (35) = -1.91, p = .064, Cohen’s d = 0.32].

**Fig 2:**
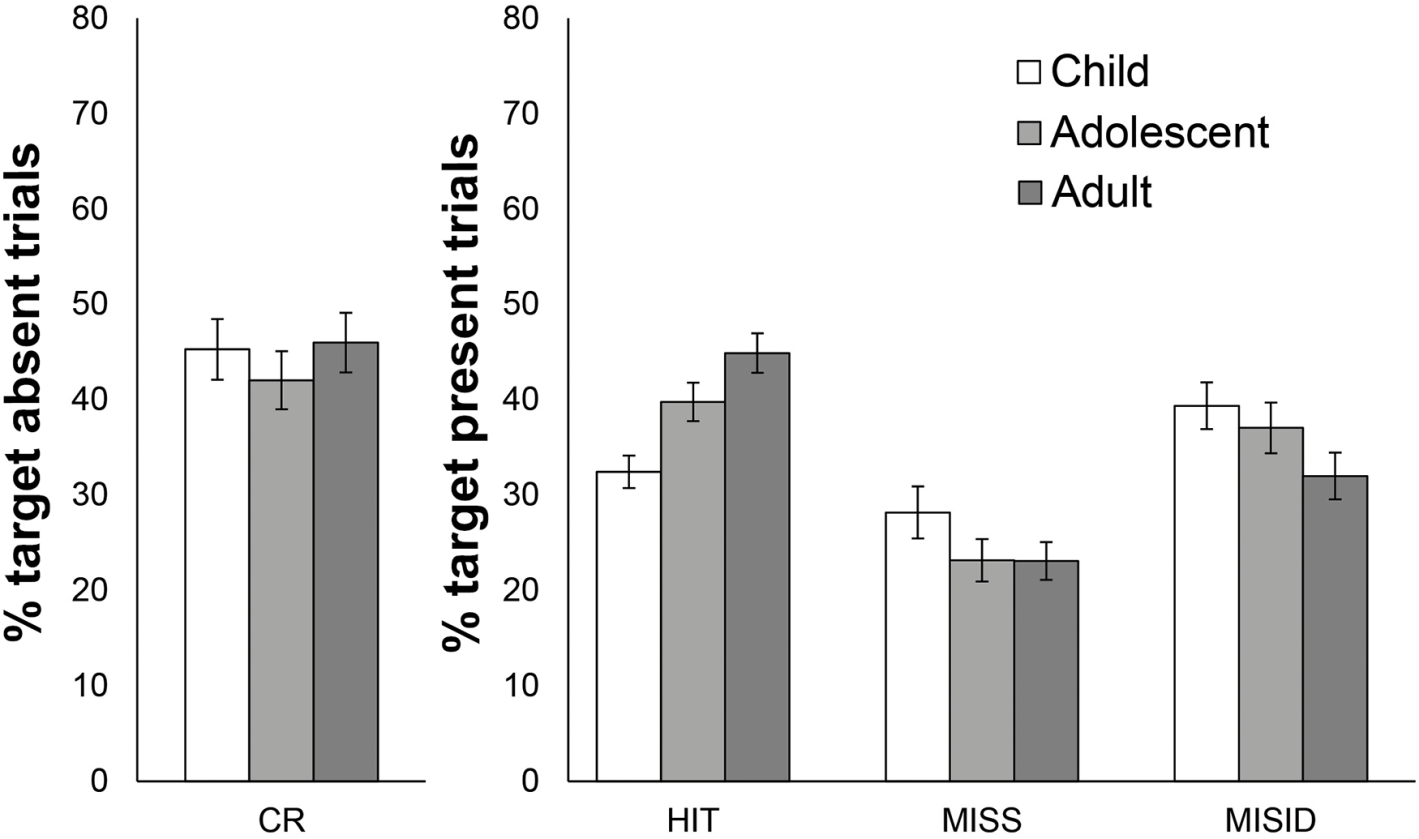
Face matching performance by age of target face in Experiment 1. Performance is expressed as correct rejection (CR), hit, miss and misidentification (MISID) rates. Error bars denote standard error.

We analysed performance separately for the following response types. For target absent trials, correct ‘absent’ responses were classified as *correct rejections* (false alarms were not analysed as they are the complement of this measure). For target present trials a *hit* response was registered when the correct identity was selected. Participants could make two types of error in target present trials: *misidentifications*, by selecting the wrong identity, and *misses* by indicating that the target is not present in the array. We analyzed the effect of target age separately for each of these four response types. Mean proportions of each response type are shown separately for each stimulus condition in Fig 2.

For correct rejections, there was a marginally significant effect of Applicant Age [F (2,70) = 2.95, p = .059, η_p_^2^ = .078], with planned comparisons confirming that participants made fewer correct rejections for adolescent compared to child [t (35) = 3.25, p < 0.05, Cohen’s d = 0.39] and adult applicants [t (35) = 3.96, p < 0.05, Cohen’s d = 0.37] but no difference between for child and adult applicants [t (35) = 0.71, p > 0.05, Cohen’s d = 0.06].

For hits, participants were less likely to find younger target identities when present in a candidate list. The main effect of Applicant Age was significant [F (2,70) = 27.15, p < 0.05, η_p_^2^ = .437], with planned comparisons confirming that participants made fewer hits for child compared to adolescent applicants [t (35) = 4.12, p < 0.05, Cohen’s d = 0.69] and for adolescent compared to adult applicants [t (35) = 3.02, p < 0.05, Cohen’s d = 0.44].

The main effect of applicant age was also significant for misses [F (2,70) = 6.70, p < 0.05, η_p_^2^ = .161] and misidentifications [F (2,70) = 11.54, p < 0.01, η_p_^2^ = .248]. Planned comparisons show more misses in child compared to adolescent [t (35) = 3.11, p < 0.05, Cohen’s d = 0.55] and adult arrays [t (35) = 2.90, p < 0.05, Cohen’s d = 0.56] but no difference between adolescents and adults (*t* < 1). For misidentifications, the difference between child and adolescent applicants was non-significant [t (35) = 1.33, p > 0.05, Cohen’s d = 0.22]. Children [t (35) = 4.75, p < 0.05, Cohen’s d = 0.79] and adolescents [t (35) = 3.63, p < 0.05, Cohen’s d = 0.62] were misidentified more often than adult applicants. Thus, lower hit rates for younger applicants was caused by an increase in both miss and misidentification errors.

### Discussion

Overall, performance on the FR candidate list task was very poor. For adult target faces, participants were incorrect on over *half* of all trials, suggesting that previous studies of unfamiliar face matching may overestimate human accuracy in a variety of modern applied settings. Error rates were especially poor for images of adolescents and children, despite the shorter average time interval between target and array images for children (6.2 years) and adolescents (6.3 years) compared to adults (9.7 years). This suggests that accelerated growth of facial structure in childhood and adolescence [24, 25] increases task difficulty for humans; as has previously been shown for machine recognition [27]. Further, consistent with the effect of face aging on matching adult faces [23], poorer performance for younger compared to older targets was only evident in target present trials.

Given the very low overall accuracy in this test, it becomes important to ask whether these levels of performance are also observed in people who use FR software in their daily work. If error rates in Experiment 1 reflect error in professional operators, realistic estimates of overall accuracy in systems employing both FR algorithms and human operators would be *half* that reported in benchmark tests of the machine component in FR systems. This result has significant implications for critical identity verification processes in forensic and security settings. However, it is possible that in workplaces where staff routinely use this software, performance is improved through experience and training. In the next study we addressed this question by comparing performance of trained passport officers to a control group of untrained students.

## Experiment 2

In Experiment 2, we tested performance of passport issuance officers on a task that modeled the FR system used by these staff their daily work. All images used in the study were real passport images, and the algorithm that selected images to be displayed in candidate lists was the same as that used to check for identity fraud in passport applications in their workplace. Because of their experience and training in this task we expected that they would outperform groups of untrained novice participants. We tested two groups of passport officers: *Facial Review* staff, and a more specialist group of *Facial Examination* staff (for definitions of these roles see [19]). Facial examiners perform more detailed facial image comparisons, receive more training and spend more time each day performing face matching tasks than facial review staff. In recent studies, facial examiners were more accurate in one-to-one facial image comparison compared to control groups [20, 21] and so we expected that examiners would outperform the other groups.

### Ethics Statement

This study was approved by the Human Research Ethics Committee at the University of New South Wales. All participants provided written informed consent and appropriate photographic release.

### Method

#### Participants

We tested two groups of passport issuance staff, and a comparison group of untrained participants. The group of 24 *Facial Review* staff (18 Females, Mean Age = 45.9, SD = 12.4) were passport issuance officers who had received limited training and whose primary role was to assess the eligibility of passport applications (similar to participants in [17]). Verifying whether a passport applicant is present in the FR candidate list is one of many tasks that make up the passport eligibility checking process. Employment duration of this group varied between 2 months to 26 years, with the mean duration being 8.2 years (SD = 6.4).

The second group of passport officers, *Facial Examiners*, were a group of 7 specialist staff (4 Females, Mean Age = 40.9, SD = 10.9) who examine facial images in cases of suspected fraud that had been flagged during the eligibility checking process (i.e. by *Facial Reviewers*). These staff spend a significant part of their working day carrying out detailed analysis of facial images, and include court-practicing forensic examiners with many years’ experience in making face matching decisions. Employment duration with the Australian Passport Office varied between 2 and 8 years (M = 4.3; SD = 2.4) and two facial examiners had additional experience (> 15 years) performing facial image comparison and document examination for other agencies. Performance of these groups were compared with performance of 48 untrained university students and members of the general public (29 Females, Mean Age = 27.2, SD = 8.9).

#### Stimuli

720 target identities were randomly selected from the passport image database using the same sampling procedure as in the previous experiment. These stimuli were used to create four versions of the matching task, with counterbalancing ensuring equal assignment of participants to stimulus sets across participant groups. As before, we included an equal number of child, adolescent and adult target identities to ensure that stimuli were representative of daily work of passport officers. However, as the primary aim of this study was to compare performance of users of face recognition software to untrained university students, we were not specifically interested in the effect of applicant age in this experiment. A list of stimulus items coded for face age and ethnicity used in this study, in addition to itemised performance measures, are provided in S1 Data.

#### Design and procedure

Design and procedure were identical to the previous experiment. However, because we tested passport officers during their normal working day—making it necessary to reduce the length of the experimental session—each participant completed only 180 trials in a random order (half target present, half target absent). In addition, we limited presentation of each stimulus array to 18 seconds for all participants; after this time had elapsed participants were forced to make a response. Trials were presented in three blocks of 60 trials with a short break provided between blocks, and the experiment lasted approximately forty minutes.

### Results

#### Group performance

**Fig 3:**
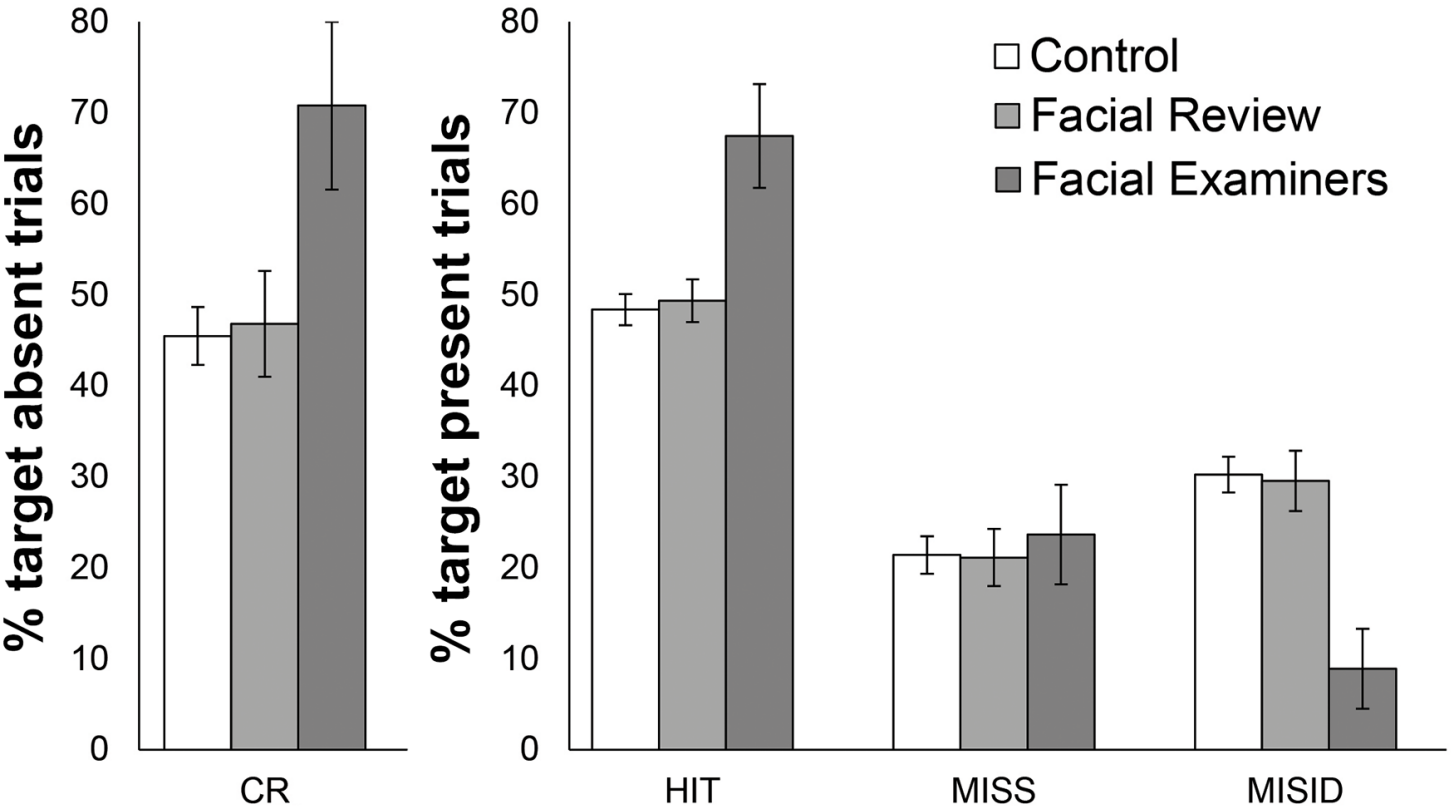
Face matching performance of participant groups in Experiment 2. Performance is expressed as correct rejection (CR), hit, miss and misidentification (MISID) rates. Error bars denote standard error.

Examiners made substantially fewer errors compared to the other groups. Overall accuracy in facial examiners (M = 69.1%, SD = 16.9%) was substantially better than both facial review staff (M = 48.1%, SD = 15.9%; t (29) = 3.04, p < 0.05, Cohen’s d = 1.13) and students (M = 47.6%, SD = 12.8%; t (53) = 4.01, p < 0.05, Cohen’s d = 1.10). The difference between review staff and students was non-significant (t < 1, Cohen’s d = 0.035). Analysis of response bias revealed no significant differences between criterion scores (F < 1) for students (M = -.09, SD = .39), facial review staff (M = -.03, SD = .46) or examiners (M = .06, SD = .33). To examine accuracy differences in greater detail, we carried out separate one-way ANOVAs for the four response types described in Experiment 1: *Correct Rejections*, *Hits*, *Misses* and *Misidentifications*. Mean proportions of each response type are shown in Fig 3.

For target absent trials there was a significant main effect of group on correct rejections, [F (2,76) = 3.37, p < .05, η_p_^2^ = .081]. As is evident from visual inspection of Fig 3, this main effect was driven by superior performance in facial examiners—who made 25% less errors in target absent trials compared with both control participants [t (53) = 2.81, p < 0.05, Cohen’s d = 0.77] and facial review staff [t (29) = 2.02, p = 0.053, Cohen’s d = 0.75], with the latter difference being marginally significant. The difference between facial review staff and control participants was non-significant [t (70) = .220, p > 0.05, Cohen’s d = 0.05].

For target present trials, there was a main effect of Group on hit responses [F (2,76) = 7.82, p < 0.05, η_p_^2^ = .171], which was again driven by examiner’s superior performance—making 18% more hits than both facial review staff and control participants [Examiners vs. Review: t (29) = 3.43, p < 0.05, Cohen’s d = 1.27; Examiners vs. Control: t (53) = 2.81, p < 0.05, Cohen’s d = 0.77; Facial Review V Control: t < 1].

Superior performance in target present trials for the examiner group was driven by a selective reduction in misidentification errors. The significant main effect of group [F (2,76) = 6.94, p < .05, η_p_^2^ = .154] was driven by 20% fewer misidentifications in examiners compared with other groups [Examiners vs. Review: t (29) = 3.12, p < 0.05, Cohen’s d = 1.16; Examiners vs. Control: t (53) = 3.94, p < 0.05, Cohen’s d = 1.08; Facial Review V Control: t < 1]. Interestingly, the main effect of Group in miss responses was non-significant (F < 1). Thus, examiners were equally likely to miss matching faces in the candidate list compared to other groups, but were less likely to select non-matching faces when the target was present.

#### Relationship between professional experience and face matching accuracy

**Fig 4:**
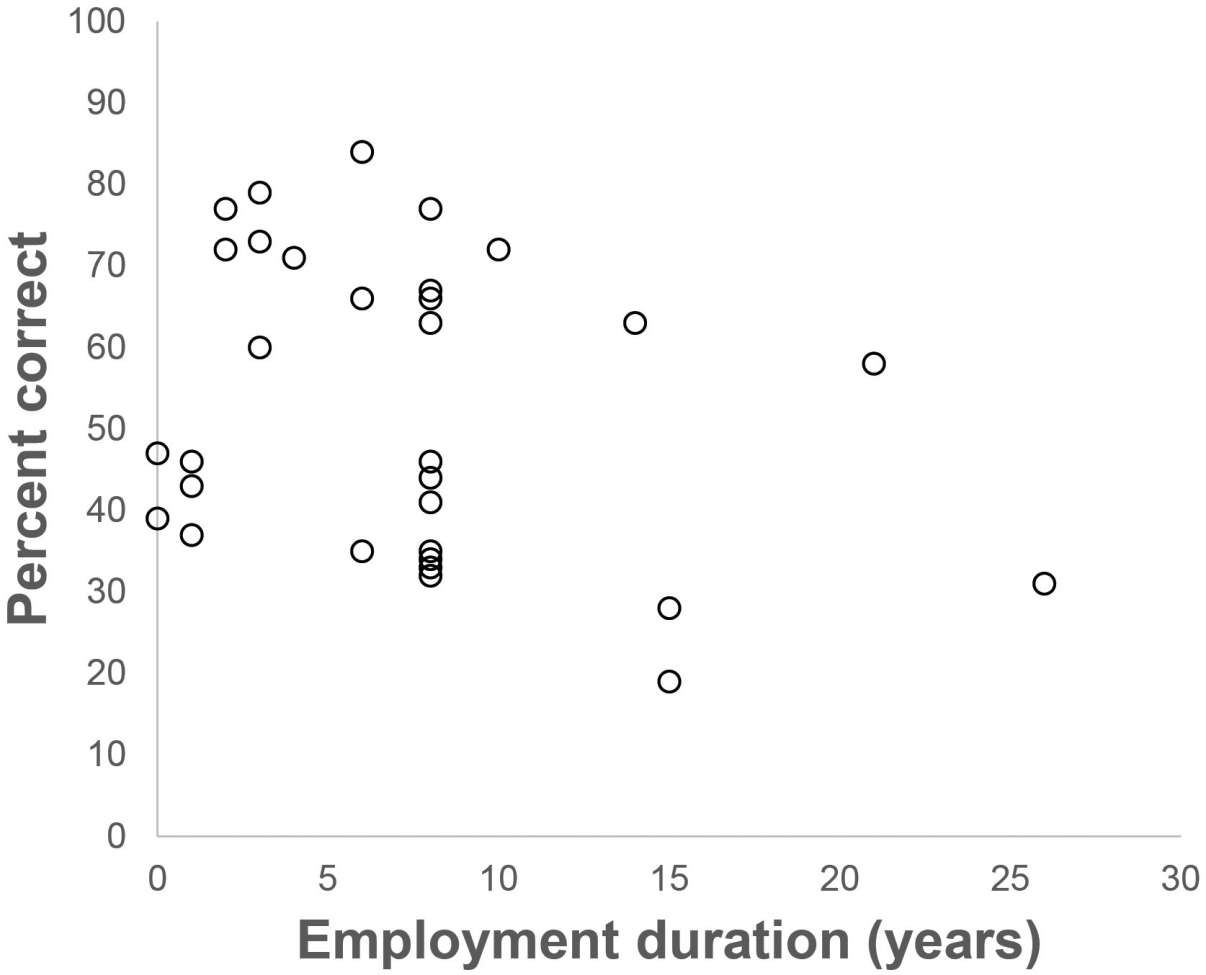
Scatterplot showing non-significant correlation between Passport Officers' employment duration and overall face matching accuracy. The graph does not differentiate facial review staff from facial examiners to protect participant anonymity.

To examine whether experience performing the FR candidate list task was predictive of accuracy, we correlated overall accuracy on the task with number of years employed at the Australian Passport Office. This relationship is plotted in Fig 4. Consistent with earlier work [17], there was no relationship between employment duration and face matching accuracy either for facial review staff [n = 24, Spearman’s rho = -.248, p > 0.05], or for facial examiners [n = 7, Spearman’s rho = .000, p > 0.05]. Although unrelated to professional experience, there was nevertheless a large range of individual differences in accuracy (ranging from 20% to 85% correct), which is also consistent with previous studies of novices [15] and passport officers [17].

#### The effect of face ethnicity on face matching accuracy

Previous studies show poorer accuracy when matching faces that are of a different ethnicity to the viewer [16]. Although we did not set out to test this, given the ethnic diversity of Australian citizens used as stimuli in this study, we conducted a post-hoc comparison of face matching accuracy for Caucasian and non-Caucasian faces. Three independent judges coded the ethnicity of target faces and we then compared item accuracy for Caucasian and non-Caucasian target subsets (see S1 Data for coding criteria, item ethnicity and itemised accuracy). Surprisingly, this analysis revealed higher overall accuracy for *non*-Caucasian targets (n = 133; M = 52.3%; SD = 19.0%) compared to Caucasian targets (n = 587; M = 48.1%; SD = 17.7%) [t (726) = 2.45, p <0.05, Cohen’s d = 0.23]. We then performed a subject-level analysis by computing face matching performance on Caucasian and non-Caucasian subsets separately for each participant. Analysis of subject performance also revealed higher accuracy on non-Caucasian compared with Caucasian target faces, driven by non-Caucasian foil faces attracting fewer False Alarm [F (1,76) = 3.92, p = .052, η_p_^2^ = .049] and Misidentification errors [F (1,76) = 10.64, p < 0.05, η_p_^2^ = .123]. All interactions between target ethnicity and group were non-significant (F < 1; for full details of this analysis see S1 Text).

This result is opposite to that reported in similar face matching studies, where other race faces attract *more* matching errors than own-race faces [16]. One possible explanation for this discrepancy is that the FR algorithm selected more confusable foils when searching for Caucasian targets compared to non-Caucasian targets. Previous studies show that accuracy of face recognition algorithms is sensitive to the demographic composition of both training and test image databases [28, 29]. Moreover, approximately three-quarters of Australian Passport holders are Caucasian, and so the foil stimuli in Caucasian target arrays were selected from a much larger database, making it more likely that the algorithm would identify highly confusable non-matching images. It is not certain that this contributed to the effect of target ethnicity here. However, FR software is often used by ethnically diverse workforces to search ethnically diverse databases, and so the nuanced interaction between ethnicity of human users, algorithms, image databases and target faces is an important topic for future work.

## General Discussion

Overall, our results show very poor face matching performance in a realistic photo-to-photo matching task based on the output of commercial off-the-shelf face recognition software. This task used real passport application images and was designed to model face matching decisions made by FR operators in a passport issuance workflow. In both experiments, untrained participants made over 50% identification errors. More importantly, trained passport officers who use this software in their daily work—making an average of 60 candidate list checks per day—were no better on the task than untrained student participants. These results have clear implications for the reliability of identity verification systems that employ human operators to monitor the output of FR software. In systems implementing one-to-many algorithm checks, it is human users that are required to make final identity decisions, and so the accuracy of the system as a whole is heavily constrained by human accuracy. Performance data reported here equate to errors on average in 1 of every 2 candidate lists. Thus, estimates of operational FR system accuracy, in the conditions we have tested here, can be computed by *halving* scores attained on recent benchmark tests [1].

This level of performance was observed despite using real passport photographs in the FR candidate list task, which are subject to strict quality control measures designed to ensure that they are truthful representations of appearance [30]. Despite these constraints, error rates were substantially higher than in previous studies of face matching performance using one-in-ten face matching arrays [12]. To account for this, and consistent with recent work [17, 23] we suggest that face aging caused poorer performance compared to similar tasks where images were taken on the same day [12]. However, face age only affected accuracy in target present trials, both here and in previous work [17, 23], and so does not account for the equivalent error rates we observed in target *absent* arrays. Comparable error rates when targets did not appear in candidate lists suggests that ‘foil candidates’–selected by the computerised FR engine from a database of millions of passport images—were more easily mistaken for the target identity than foils selected by humans from much smaller databases in previous studies [12]. Therefore the capability of FR software to search enormous databases of facial images comes at a cost: it significantly increases the difficulty of the task faced by the human operator.

It is important to note that the task performed by participants in this study, although specifically designed to estimate accuracy rates in users of FR systems, differs from operational conditions. To derive reliable measures of perceptual performance in the limited time we had to test passport officers, target images appeared in candidate lists on half of trials. In many operational settings where the task is to find a matching face in an array—such as when using FR software to search for identity fraud in passport applications—it is very rare that target identities appear in candidate lists. This is because base rates of identity fraud are very low, with some reports estimating 1 in 400 passport applications to be fraudulent [18]. Estimating match detection accuracy at these prevalence rates presents a practical challenge. However, lab based studies that model the task of checking photo-ID for secure access show that non-match prevalence rates of 10% lead to poorer detection accuracy [31] suggesting that performance in operational conditions may be substantially poorer than reported here. Future work that measures match detection in the workplace, perhaps by inserting known matches into candidate lists, may be necessary to provide accurate estimates of user error in face recognition systems [2].

Encouragingly, specialist facial examiners performed far better on the candidate list task than untrained students and the non-specialist passport officers—making 20% fewer errors as a group. However, as is typical in studies of expert populations [32], our data do not isolate the factors that cause their superior performance. One potential cause is that examiners were naturally better at the task. This would be consistent with previous studies showing that some people perform very well in face matching tasks while others perform very poorly [15, 33, 34] and that these individual differences are stable across tests [33–35].

However, at the time this experiment was carried out, the Australian Passport Office did not use formal selection processes to recruit facial reviewers, nor to select facial examiners. However, it is possible that informal selection processes occurred. For example examiners may have been motivated to continue in their role because they believed they were good at the task. Alternatively, recent work has shown that providing feedback on the accuracy of face matching decisions [36, 37] and performing face matching decisions in groups [38] can improve accuracy in face matching tasks. Therefore, by working alongside other facial examination specialists, receiving feedback and discussing their work, examiners may also have developed more effective strategies for face matching. Future work should aim to clarify precisely what aspects of training and experience cause improvement in performance on face matching tasks. Whilst guidelines for training in facial comparison do exist [19, 39, 40], these have not yet been subject to empirical validation. Indeed, some recommended methods have been shown to be ineffective in improving accuracy [41, 42].

Notwithstanding, trained facial examiners have outperformed control participants both here and in recent studies [20, 21]. Further, differences between facial forensic examiners and standard experimental groups are not limited to quantitative differences in overall accuracy–*qualitative* differences also point towards differences in the cognitive processing they deploy. In the present study we found that superior performance in examiners resulted from a reduction in false alarm and misidentification errors (i.e. choosing errors). Examiners made the same proportion of *miss* responses as other groups (i.e. non-choosing errors), raising the possibility that their expertise is tuned towards detecting differences between faces. More generally, this result builds on recent reports of other qualitative differences in performance of forensic facial examiners. For instance, expertise of examiners is most evident at longer exposure durations [20] and when comparing high-quality images [21]. Examiners also show less impairment by image inversion [20] in comparison to university students. These qualitative differences offer important insights into the nature of expertise that can be used to inform development of training methods and workflow systems for facial image comparison.

Finally, given the very clear limits that human performance imposes on the reliability of systems employing ‘automatic’ face recognition software, computer scientists and psychologists should work together to improve total system accuracy. Studies reported here and in previous work show large and stable individual differences in face matching accuracy that are unrelated to length professional experience [15, 17, 32]. We therefore propose that selection of staff based on face matching performance can provide substantial gains in the reliability of FR systems at little cost. Testing staff on face recognition workflow simulations, as we have here, enables selection tests to be tailored to the precise task demands faced by users in daily work and to monitor their performance over time. Technical solutions that build human testing capabilities into face recognition software may therefore be useful in selecting individuals best suited to operating this software, and in evaluating training and mentorship programs. More broadly, face recognition software developers, user experience designers and system administrators should give careful consideration to the abilities of human operators when designing face recognition systems.

## Supporting Information

S1 Data Subject and item data. Excel spreadsheet containing performance data from Experiments 1 and 2, separately for each participant (by-subject) and for each target identity (by-item). Target identities used in Experiment 2 are coded for ethnicity.
(XLSX)

S1 Text Analysis of target face ethnicity. Details of post-hoc analysis of face matching accuracy as a function of target ethnicity in Experiment 2.
(PDF)
