# Supplementary material for: Error Rates in Users of Automatic Face Recognition Software
Source: PLoS One. 2015 Oct 14;10(10):e0139827. doi: 10.1371/journal.pone.0139827 (PMC4605725; doi:10.1371/journal.pone.0139827)
Supplement: S1 Text — Details of post-hoc analysis of face matching accuracy as a function of target ethnicity in Experiment 2. (PDF) [file pone.0139827.s002.pdf]

“Error rates in users of automatic face recognition software” White et al.

S1 Text. Analysis of target face ethnicity (Experiment 2)

Because Australia has an ethnically diverse population, we carried out a post-hoc analysis of the effect of face ethnicity on face matching performance (see Results section of Experiment 2 in the manuscript for discussion). First, three independent judges coded the ethnicity of target faces (see S1 Data for coding criteria). For each participant, we then calculated proportions of each response type, separately for Caucasian and non-Caucasian target faces. Summary statistics are shown in Table S1 (next page). These data were analysed separately for each response type, by 2x3 mixed factor ANOVA with Target Ethnicity (Caucasian, Other) and Group (control, facial review, facial examiner) as factors. Results of this analysis were as follows:

Correct Rejections. The main effect of Target Ethnicity was marginally significant [$F (1,76) = 3.92$, $p = .052$, $\eta_p^2 = .049$], with higher accuracy for non-Caucasian compared to the Caucasian faces. The interaction between Target Face and Group was non-significant [$F (2,76) = 0.99$, $p > 0.05$, $\eta_p^2 = .025$].

Hits. The main effect of Target Ethnicity and interaction between Target Ethnicity and Group were non-significant [Target Ethnicity: $F (1,76) = 1.17$, $p > 0.05$, $\eta_p^2 = 0.15$; Interaction: $F (2,76) = 0.24$, $p > 0.05$, $\eta_p^2 =.006$].

Misses. The main effect of Target Ethnicity and interaction between Target Ethnicity and Group were non-significant [Target Ethnicity: $F (1,76) = 2.83$, $p > 0.05$, $\eta_p^2 = 0.36$; Interaction: $F (2,76) < 0.1$, $p < 0.05$, $\eta_p^2 = .000$].

Misidentifications. There was however, a main effect of race for misidentification errors [$F (1,76) = 10.64$, $p < 0.05$, $\eta_p^2 = .123$]. Surprisingly, we found that misidentification errors to be higher for Caucasian compared to other race faces. The interaction with group factor was not significant [$F (2,76) = .36$, $p > 0.05$, $\eta_p^2 = .009$].

	**Caucasian Targets**				**non-Caucasian Targets**			
	CR	*Hits*	*Miss*	*MisID*	*CR*	*Hits*	*Miss*	*MisID*
Control	43.3 (23.1)	47.2 (13.7)	19.8 (15.0)	33.1 (15.8)	44.7 (24.9)	50.6 (14.8)	22.6 (17.4)	26.8 (16.0)
FR Reviewers	45.7 (28.5)	48.7 (11.9)	20.7 (15.6)	30.7 (15.8)	51.6 (30.0)	52.3 (12.9)	23.4 (16.6)	24.3 (19.8)
FR Examiners	69.9 (23.9)	67.4 (15.4)	23.1 (14.9)	9.5 (12.4)	74.6 (27.1)	67.1 (15.7)	26.2 (15.1)	6.71 (9.4)

Table S1. Mean response rates for each response type, separately for Caucasian and non-Caucasian faces, for each participant group in Experiment 2.
